# Does dystonic muscle activity affect sense of effort in cervical dystonia?

**DOI:** 10.1371/journal.pone.0172019

**Published:** 2017-02-13

**Authors:** Loïc Carment, Marc A. Maier, Sophie Sangla, Vincent Guiraud, Serge Mesure, Marie Vidailhet, Påvel G Lindberg, Jean-Pierre Bleton

**Affiliations:** 1 FR3636, CNRS / Université Paris Descartes, Sorbonne Paris Cité, Paris, France; 2 Université Paris Diderot, Sorbonne Paris Cité, Paris, France; 3 Service de Neurologie, Fondation OPH de Rothschild, Paris, France; 4 Université Paris Descartes, Sorbonne Paris Cité, INSERM U894, Paris, France; 5 Service de Neurologie et Unité Neurovasculaire, Hôpital Sainte-Anne, Paris, France; 6 UMR 7287, CNRS Aix Marseille Université, Institut des Sciences du Mouvement, Marseille, France; 7 Sorbonne Universités, UPMC Univ Paris 06, UMR S 1127, Paris, France; 8 AP-HP, Hôpital de la Pitié Salpêtrière, Département de Neurologie, Paris, France; 9 Centre de Psychiatrie et Neurosciences, Inserm U894, Paris, France; University of Florida, UNITED STATES

## Abstract

**Background:**

Focal dystonia has been associated with deficient processing of sense of effort cues. However, corresponding studies are lacking in cervical dystonia (CD). We hypothesized that dystonic muscle activity would perturb neck force control based on sense of effort cues.

**Methods:**

Neck extension force control was investigated in 18 CD patients with different clinical features (7 with and 11 without retrocollis) and in 19 control subjects. Subjects performed force-matching and force-maintaining tasks at 5% and 20% of maximum voluntary contraction (MVC). Three task conditions were tested: i) *with* visual force feedback, ii) *without* visual feedback (requiring use of sense of effort), iii) without visual feedback, but with neck extensor *muscle vibration* (modifying muscle afferent cues). Trapezius muscle activity was recorded using electromyography (EMG).

**Results:**

CD patients did not differ in task performance from healthy subjects when using visual feedback (ANOVA, p>0.7). In contrast, when relying on sense of effort cues (without visual feedback, 5% MVC), force control was impaired in patients without retrocollis (p = 0.006), but not in patients with retrocollis (p>0.2). Compared to controls, muscle vibration without visual feedback significantly affected performance in patients with retrocollis (p<0.001), but not in patients without retrocollis. Extensor EMG during rest, included as covariate in ANOVA, explained these group differences.

**Conclusion:**

This study shows that muscle afferent feedback biases sense of effort cues when controlling neck forces in patients with CD. The bias acts on peripheral or central sense of effort cues depending on whether the task involves dystonic muscles. This may explain why patients with retrocollis more accurately matched isometric neck extension forces. This highlights the need to consider clinical features (pattern of dystonic muscles) when evaluating sensorimotor integration in CD.

## Introduction

Cervical dystonia (CD) is clinically characterized by involuntary neck muscle contraction leading to abnormal movement or posture [1]. Integration of multimodal sensory information is necessary to accurately execute voluntary movements. This integration seems to be deficient in CD [2]. Similarly, controlling forces using sense of effort cues are affected in focal dystonia [2,3]. Sense of effort contributes to the control of body position, forces and movements and includes central cues (efferent copy: derived from the motor command) as well as peripheral cues (from muscle afferents) [4]. It has been suggested that involuntary neck muscle contractions may perturb the peripheral contribution to the sense of effort in CD [1]. However, despite potential interest for targeted rehabilitation approaches, studies investigating the relation between clinical features of CD, neck control and sense of effort are lacking.

Kinematic studies have shown that in CD neck extension amplitude and velocity are reduced toward the non-dystonic side (anti-dystonic) in voluntary movements compared to movements toward the dystonic side (pro-dystonic) [5,6]. These results were explained by more efficient muscle activation patterns (muscle synergies) during pro-dystonic movements [5]. During anti-dystonic movements, the overflow phenomenon, i.e., a lack of muscle selectivity often described in focal dystonia [7], was thought to impede appropriately timed relaxation of dystonic muscles [8]. Thus, involuntary muscle activity reduces specificity of task-related sense of effort cues.

Furthermore, even though anti-dystonic movements are impaired in all planes, movement control in the sagittal plane, i.e. in flexion-extension, may be more severely affected [5,6].

Together these previous studies suggest that control of neck extension movements and forces will differ depending on clinical features, i.e. on whether task-related muscles are dystonic or not. This leads to the prediction that neck control involving dystonic muscles will be less affected than neck control involving non-dystonic muscles in patients with CD. Particularly, in CD flexion-extension afferent feedback will be differentially affected by the presence or absence of retrocollis. The underlying rationale is that the reliability of sensorimotor information processing [9] depends in part on muscle afferent feedback.

We hypothesized that multisensory integration (of visual and sense of effort cues) during voluntary isometric neck force control would be differentially affected in CD patients with varying clinical features (i.e., presence or absence of retrocollis).

## Methods

### Participants

Eighteen patients with primary focal CD were recruited and categorized according to clinical features, i.e. presence (CD_R+, N = 7) or absence (CD_R-, N = 11) of a retrocollis. Patients with tardive/drug-induced dystonia were excluded. None of the patients received botulinum toxin injections for at least 3 months prior to this study. Nineteen healthy (age- and gender-matched) control subjects were also recruited (Table 1: demographic and clinical details). The study received ethical approval from the regional ethics committee (Ile de France VIII) and all subjects provided written informed consent.

**Table 1:** Demographic and clinical data. Clinical data for patients with a retrocollis (CD_R+) and without a retrocollis (CD_R-). The only significant difference between groups was lower neck extension MVC in CD_R+ patients compared to the control group (p<0.019). Scores of the clinical scales did not differ between CD_R+ and CD_R- patients. There was no difference in the demographic data between groups.

CD_R-	Gen-der	Age (yrs)	MVC (N)	Neck circum. (cm)	Duration of CD (m)	Last BoNT inj.	PTT (visits/wk)	TWSTRS			
								Total	Severity	Disability	Pain
1	M	32	62	40	2	12	8	47	22	12	13
2	F	60	58	35	18	6	0	8	4	1	3
3	F	70	57	35	15	4	1	28	18	1	9
4	F	66	53	33	7	6	8	35	19	11	5
5	M	52	62	42	5	NT	0	23	9	4	10
6	F	45	44	33	6	3	1	42	22	8	12
7	M	64	57	36	7	6	1	27	15	9	3
8	F	65	53	34	3	9	1	25	14	9	2
9	F	49	44	34	2	NT	0	19	18	1	0
10	F	39	44	34	0	NT	0	24	17	7	0
11	F	57	40	33	12	NT	0	18	15	1	2
Mean ±SD	8F, 3M	54.45 ±12.18	52.18 ±7.90	35.36 ±2.98	7 ±5.74	6.57 ±3.05	1.82 ±3.09	26.91 ±11.07	15.73 ±5.37	5.82 ±4.33	5.36 ±4.78
CD_R+											
1	F	69	48	34	8	4	1	49	21	15	13
2	M	43	48	42	9	6	1	18	7	4	7
3	F	65	40	40	4	5	1	43	25	10	8
4	F	56	35	35	2	4	0	43	22	11	10
5	F	38	40	29	3	12	0	16	10	2	4
6	M	29	40	35	4	9	1	34	21	8	5
7	M	41	57	43	0	NT	0	47	22	15	10
Mean ±SD	4F, 3M	48.71 ±14.86	**44 ±7.42**	36.86 ±5.01	4.29 ±3.20	6.67 ±3.20	0.57 ±0.53	35.71 ±13.63	18.29 ±6.87	9.29 ±5.02	8.14 ±3.13
Control subjects											
Mean ±SD	12F, 7M	52.74 ±13.76	54.32 ±10.73	35.59 ±3.39	—	—	—	—	—	—	—

Abbreviations: TWSTRS = Toronto Western Spasmodic Torticollis Rating Scale; Yrs = years; N = Newton; circum = circumference; m = months; BoNT inj. = time (in months) of last botulinum neurotoxin injection; NT = non treated, PTT = Physical therapy treatment according to standardized physical therapy program including retraining of neck movements and posture [10], wk = week.

### Clinical assessments

Dystonic symptoms were assessed with the Toronto Western Spasmodic Torticollis Rating Scale (TWSTRS) [11]. Maximum voluntary neck extension force (MVC) was recorded using a dynamometer (Biometrics, France) applied to the back of the head.

### Neck extension tasks

Two tasks were developed (using Spike2/CED1401, http://ced.co.uk) to investigate multi-modal sensory processing during isometric force control.

**Fig 1:**
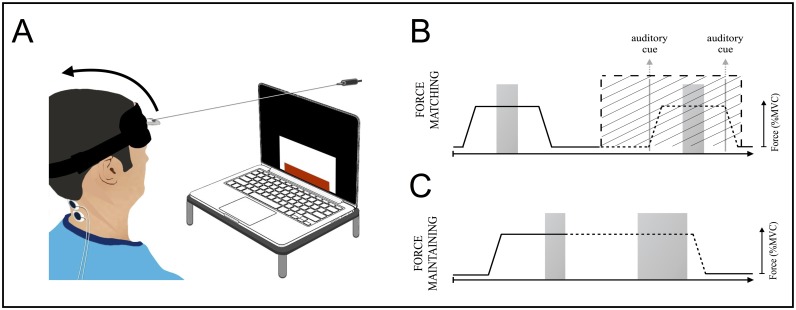
Setup and visuomotor tasks. (A) Setup for visuomotor tasks. Subjects were seated in front of a computer screen. A headband was attached to the force sensor by a non-extensible wire. The task consisted of a series (trials) of visually displayed target forces (height of white rectangle) to be matched as closely as possible using visual feedback of the exerted neck force (height of red rectangle). (B) Force matching task: subjects matched the neck extension force to an indicated target level (5% or 20% MVC) with visual force feedback (*condition_Vis*) and reproduced the same force level without visual feedback (*condition_NoVis*). In conditions without vision, subjects were given an auditory cue indicating force onset, offset or hold. Five trials/condition were presented in a pseudo-randomized order. Force exerted during the stable part of the hold phase, indicated by grey shading, was analyzed. (C) Force-maintaining task: subjects maintained their extension force at target level with visual feedback (*condition_Vis*). The visual feedback was then removed for six seconds (*condition_NoVis*) and vibration was applied (*condition_NoVis+Vib*).

**Fig 2:**
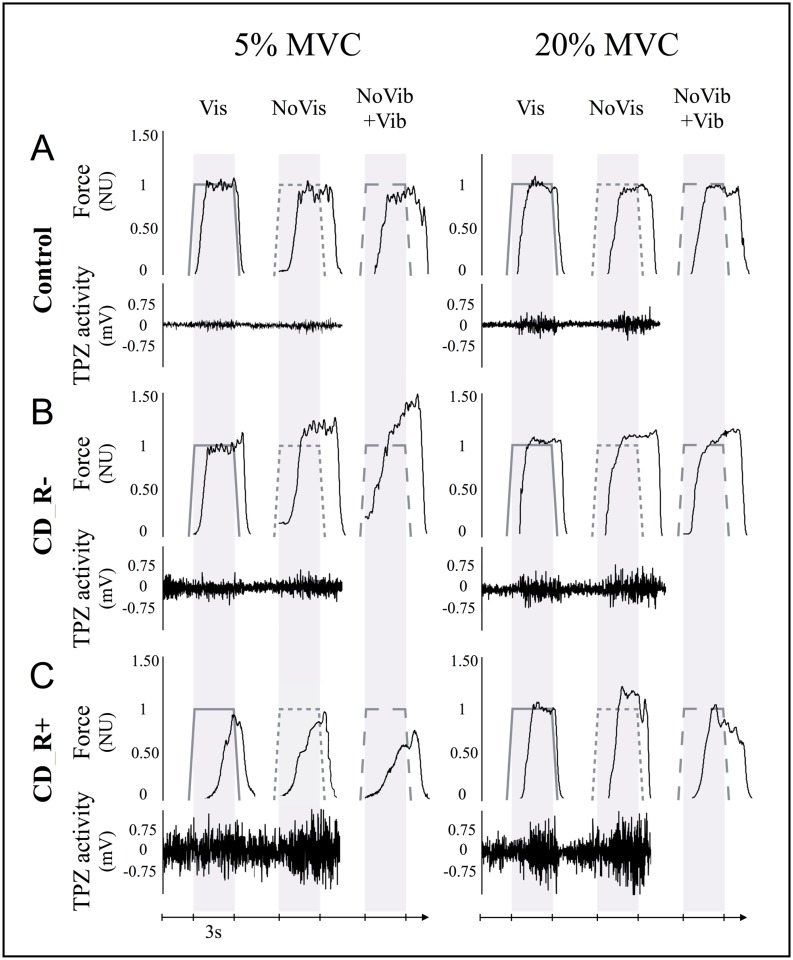
Comparison of raw data for a control subject, a CD_R- and a CD_R+ patient. (A) Control subject: raw data recorded during the force matching task at 5% MVC and 20% MVC: Neck extension force was first down-sampled (100Hz) and normalized for each subject to the target force level (NU: normalized units). Lower trace: EMG activity of right trapezius (TPZ). Example trials show force and EMG traces during *condition_Vis*, *condition_NoVis*, and *condition_ NoVis+Vib*. Note: EMG was not recorded during vibration. (B) CD_R- patient: corresponding examples. (C) CD_R+ patient: corresponding examples.

1. *A force-matching task* (Fig 1A and 1B) was used to assess performance of voluntary neck force modulation at 5% and 20% MVC under three different task conditions:
  - *Condition_Vis*: subjects had to match their neck extension force, displayed in real-time on a screen (i.e. *with visual feedback*, *Vis*), to a visually indicated target level. In each trial, force had to be increased to target force level, then maintained (for 3s), and finally released.
  - *Condition_noVis*: subjects had to reproduce the previous trial *without visual feedback (noVis)*. This required the use of sense of effort cues to match performance between trials.
  - *Condition_noVis+Vib*: this condition was similar to condition b, but with *muscle vibration (+Vib)* to modify muscle afferent feedback [12] (70Hz vibration on the left and right trapezius muscles; Vibrasens^®^ VB115, www.technoconcept.fr).
2. *A force-maintaining task* was used to assess the ability to maintain steady neck extension force at 5% and 20% MVC (Fig 1C) [13]. This task was performed in the same three task conditions as the *Force-matching task*.
  Left and right trapezius activity was recorded using surface electromyography (EMG) (www.grasstechnologies.com; Fig 2A–2C). EMG from five subjects was not exploitable, due to either poor signal to noise ratio (N = 2) or technical failure (N = 3).

### Data analysis and statistics

Raw data was analyzed using MatlabV8.6 (The MathWorks, Inc., Natick, MA, USA) and statistics performed under Statistica10 (StatSoft, Inc., USA). Forces and EMGs were averaged across all trials in each task condition. Group differences were analyzed using a general linear model repeated measures ANOVA with one GROUP factor (CD_R-/CD_R+/Controls) and two within-group factors: FORCE (5%/20% MVC) and CONDITION (a/b/c). We used Fisher LSD to test for post-hoc differences. The level of significance was set to p<0.05 and adjusted in order to correct for multiple comparisons with the Benjamini and Hochberg method [14]. All reported p values <0.05 met corrected levels of significance.

## Results

### Isometric force-matching and force-maintaining

The two tasks were completed successfully by all subjects. Patient groups did not differ in total TWSTRS (Mann-Whitney U Test p = 0.23). Fig 2A–2C shows examples of the isometric force-matching task.

**Fig 3:**
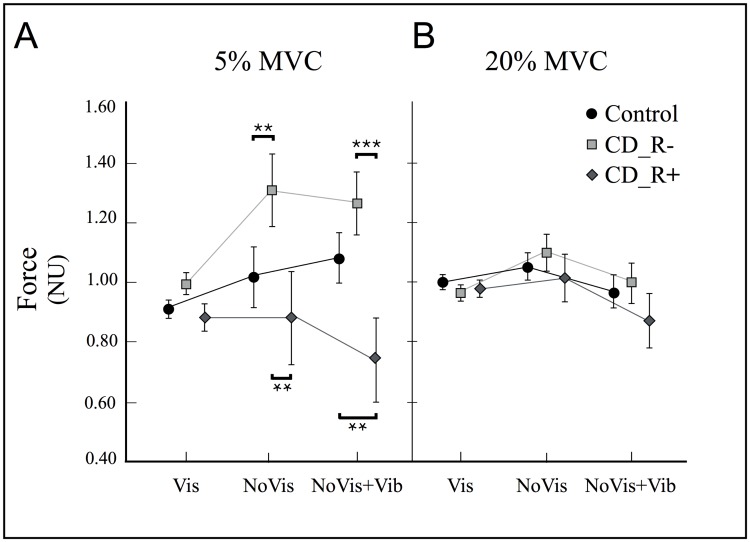
Group performance in the visuomotor force-matching task. Mean force (mean±SD) for the three groups during *condition_Vis*, *condition_NoVis* and *condition_NoVis+Vib* at 5% MVC (A) and at 20% MVC (B). (A) No significant difference in mean force between groups in *condition_Vis*. Significant differences at 5% MVC during *condition_NoVis* between CD_R- and control subjects (p = 0.006), and also between CD_R- and CD_R+ patients (p = 0.002). Note that the variability of mean force (SD) increased about 5-fold in all three groups. Significant differences during *condition_NoVis+Vib* between CD_R+ and control subjects (p = 0.006) and also between CD_R+ and CD_R- patients (p<0.001). (B) No difference in mean force at 20% MVC. * = p<0.05; ** = p<0.01; *** = p<0.001.

In the force-matching task (Fig 3A and 3B) the ANOVA of force showed a significant GROUP difference (F = 3.63, p = 0.04). This GROUP difference interacted with FORCE (F = 4.96, p = 0.01); no other interaction was found. Post-hoc testing revealed that neck extension forces were similar in patients and control subjects when using visual cues (*condition*_*Vis*, p>0.7) and GROUP differences were specific to 5% MVC-level (p<0.001 between CD groups).

However, performance of CD patients differed in conditions requiring sense of effort cues: in *condition*_*noVis* (5% MVC), force was significantly increased in CD_R- patients (1.31±0.47N) compared to control subjects (1.06±0.43N, p = 0.006), but not in CD_R+ patients (0.89±0.32N, p = 0.27). Thus, CD_R- patients applied significantly higher forces than CD_R+ patients (p = 0.002). Control subjects showed no significant force difference between *condition*_*Vis* (0.92±0.09N) and *condition*_*noVis (*1.07±0.43N, p>0.05).

During muscle vibration (*condition*_*noVis+Vib*, 5% MVC), CD_R+ patients showed significantly reduced mean force (0.74±0.33N) compared to CD_R- patients (1.26±0.35N, p<0.001) and to control subjects (1.08±0.37N, p = 0.006). Hence, compared to control subjects, CD_R- patients tended to overshoot, whereas CD_R+ patients undershot target forces.

The above ANOVA was repeated with baseline EMG activity (during rest) as covariate. This cancelled the statistical main (GROUP, p = 0.3) and post-hoc differences between CD patients and control subjects.

In the force-maintaining task, the performance of CD_R- and CD_R+ patients were qualitatively similar to those seen during force-matching. Similar between group differences were found: (i) *condition*_*Vis*: no significant difference (p>0.2); (ii) *condition*_*noVis*: force CD_R- > force controls (p = 0.003); (iii) *condition*_*noVis +Vib*: force CD_R- > force CD_R+, (p = 0.001).

### Electromyography

EMG activity during MVC was similar between groups (F = 0.69, p = 0.51). However, during force-matching, the ANOVA of EMG activity showed significant effects of GROUP (F = 10.52, p<0.001) and FORCE (F = 92.63, p<0.001), but not of CONDITION (F = 0.27, p = 0.60). Post-hoc analyses showed increased EMG activity in CD patients compared to control subjects (p<0.01). EMG activity was higher in CD_R+ than in CD_R- patients (p = 0.04).

## Discussion

We have shown that clinical features of CD (presence/absence of a retrocollis) differentially affect multisensory integration of visual and sense of effort signals during voluntary neck force control. We have also shown that the type of force control deficit depends on the characteristics and sources of sensory information (with/without vision; with/without perturbed muscle afferents). Our results are in line with the optimal multisensory integration theory, [9] which postulates that to control movement, subjects (through mechanisms of gating/weighting) rely on the most reliable information available among multi-modal sensory feedback.

With visual force feedback, performance of CD patients was similar to that of control subjects. CD patients presumably used the most reliable sensory modality (vision) and gated less reliable feedback. This is consistent with previous findings [15,16] and with the theory of optimal use of multisensory cues [9].

Without visual feedback, subjects were required to match forces (from the previous trial) by using sense of effort cues exclusively. The variability around the average force increased for all subjects. However, force control was impaired only in CD patients without retrocollis, since CD_R- patients overshot, whereas CD_R+ patients and control subjects showed no change. These results suggest that CD_R- and CD_R+ patients optimized their use of sense of effort cues differently. Presumably, CD_R+ patients favoured peripheral cues since voluntary activation of dystonic task-related muscles helped keeping agonist afferent feedback reliable. CD_R- patients may have chosen central cues since non-agonist dystonic muscles may have produced sensory afferent crosstalk, rendering the efferent copy more reliable. Baseline EMG explained group differences suggesting that spontaneous (dystonic) neck muscle activity at rest can account for modified sense of effort.

Modifying peripheral sensory cues (by vibration of neck extensor muscles) clearly affected CD_R+, but not CD_R- patients. The fact that muscle vibration acts directly on peripheral cues corroborates our assumption that CD_R- patients relied more on central sense of effort cues (not or less affected by vibration), and that CD_R+ patients relied more on peripheral sense of effort cues (strongly affected by vibration). Moreover, target forces were overshot by CD_R- patients and undershot by CD_R+ patients. This is consistent with vibration acting on dystonic agonist muscles in CD_R+ and on non-dystonic agonists in CD_R- patients. Dystonic muscles are more sensitive to vibration than non-dystonic muscles [17] and provide an over-proportional muscle afferent feedback in CD_R+ patients. This could explain why CD_R+ patients overestimated forces (and undershot the target given their over-proportional feedback). In contrast CD_R- patients, less affected by muscle vibration, underestimated forces (and overshot the target), as they did in the condition ‘without visual feedback’.

Lastly, we confirm that in focal dystonia, modulating voluntary force according to sense of effort cues is affected [2]. CD patients showed deficits at 5% but not at 20% MVC, consistent with dystonia affecting motor control specifically at low forces [2,18]. This is consistent with dystonic muscle activity being proportionally greater relative to voluntary EMG activity at low force levels. Hence, dystonic muscle activity presumably disrupts peripheral cues to a greater extent at low force levels (more sensory cross-talk). The involuntary movements contributing to this deficit in force control may result from deficient cortical and subcortical gating mechanisms: [19–21] thalamocortical connectivity patterns vary in dystonic patients with different symptoms, providing a mechanistic rationale for dystonic phenotype [22,25]. A cerebellar component cannot be excluded either [25].

Further studies in CD are needed to investigate whether our findings can be generalized to movements other than flexion-extension, such as rotational neck movements. Although this study is limited by a relatively small sample size and lack of antagonist EMG, our findings suggest that modifying sense of effort through training or neuromodulation may be a useful therapeutic approach in CD [19,23,24].

In conclusion, we found impaired voluntary neck force control in CD patients, when successful task completion required the use of sense of effort cues. Our results showed that this impaired control may be explained by altered muscle afferent feedback related to dystonic muscle contractions, which in turn may hamper optimal use of multi-modal sensory information [9], including sense of effort. In particular, our data suggest that peripheral sense of effort cues alter sensorimotor integration in CD [25]. Our findings highlight the need to consider clinical features when investigating sensorimotor control in CD.
